# *Lactobacillus johnsonii* L531 Alleviates the Damage Caused by *Salmonella* Typhimurium via Inhibiting TLR4, NF-κB, and NLRP3 Inflammasome Signaling Pathways

**DOI:** 10.3390/microorganisms9091983

**Published:** 2021-09-17

**Authors:** Shiyan Chen, Yanan Li, Bingxin Chu, Lanxin Yuan, Ning Liu, Yaohong Zhu, Jiufeng Wang

**Affiliations:** College of Veterinary Medicine, China Agricultural University, Beijing 100193, China; chen_shiyan1993@163.com (S.C.); 13796685756@163.com (Y.L.); barrylao@163.com (B.C.); yuanlanxin914@163.com (L.Y.); nliu2224@163.com (N.L.); zhu_yaohong@hotmail.com (Y.Z.)

**Keywords:** *Salmonella* Typhimurium, *Lactobacillus johnsonii*, TLR4, NF-κB, NLRP3 inflammasome, tight junctions

## Abstract

*Salmonella* Typhimurium (*S.* Typhimurium) is an aggressive zoonotic pathogen that causes enteritis and diarrhea. Antibiotic therapy is still the primary method at present. However, the increasing emergence of multi-drug resistant bacteria weakens the therapeutic efficacy of antibiotics. Probiotics have been widely studied as an alternative antibiotic therapy. In this study, we established an IPEC-J2 cell model of *S.* Typhimurium infection, aiming to determine the protective effect of *Lactobacillus johnsonii* L531 (*L. johnsonii* L531) on *S.* Typhimurium infection. As our data showed, *S.* Typhimurium infection resulted in a robust inflammatory response demonstrated by promoted protein levels of the inflammatory-related pathway (TLR4, MyD88, p-IκBα, and p-p65), increased cytokine levels of IL-6, IL-1β, IL-18, and TNF-α, and activated the NLRP3 inflammasome via promoting its assembly. However, *L. johnsonii* L531 pre-incubation inhibited the activation of the above inflammatory signaling pathways and reduced the expression levels of pro-inflammatory cytokines. In addition, *L. johnsonii* L531 alleviated the damage of *S.* Typhimurium to tight junctions ZO-1, Occludin, and Claudin-1. In summary, our findings suggested that *L. johnsonii* L531 alleviated *S.* Typhimurium-induced tight junction injury by inhibiting the TLR4/NF-κB/NLRP3 inflammasome signaling pathway.

## 1. Introduction

*Salmonella* Typhimurium (*S.* Typhimurium), a Gram-negative bacterium that replicates in cells, is an important zoonotic pathogen that causes intestinal infectious diseases worldwide [1]. Meanwhile, as one of the vital pathogens causing diarrhea in weaned piglets, *S.* Typhimurium can persist in weaners for a prolonged period (even over 28 weeks) and lastingly affect the growth performance of piglets [2]. Currently, antibiotic treatment remains the principal methodology to deal with *Salmonella* infection [3]. However, the results of epidemiological investigations indicated that multi-drug resistant *Salmonella* became more common in swine farms, and the therapeutic efficacy of antibiotics has been undermined [4].

Probiotics as a potential alternative to antibiotics have been extensively studied. A meta-analysis (63 trials, which included 8014 people) showed that feeding probiotics could shorten the duration of diarrhea [5]. Studies proved that certain strains of *Lactobacillus johnsonii* exhibited anti-inflammatory properties and attenuated systemic pro-inflammatory immune responses [6]. Our previous experiments proved that *Lactobacillus johnsonii* L531 (*L. johnsonii* L531) reduced pathogen load and intestinal inflammation in piglets challenged by *Salmonella* Infantis [7]. However, the exact mechanism by which *L. johnsonii* L531 protects the host against intestinal pathogens remains to be further elucidated.

The intestinal epithelial barrier, consisting of intestinal epithelial cells (IECs) and tight junctions, separates the microorganisms from the sterile lamina propria in the intestinal lumen, maintaining the intestinal balance and avoiding infection [8]. When infection occurs, IECs can identify unique components of pathogenic bacteria and trigger downstream anti-infective inflammatory pathways [9]. For instance, TLR4, located on the surface of IECs, recognizes lipopolysaccharide and, then, initiates the rapid activation of NF-κB, thereby promoting the synthesis of pro-inflammatory cytokines TNF-α, IL-1β, and IL-6 [10]. Furthermore, activation of NF-κB enhances the transcription of NLRP3 [11]. The NLRP3 inflammasome, an essential part of the immune system, responds to microbial infection by mediating the activation of Caspase-1 and the secretion of pro-inflammatory cytokines IL-1β/IL-18 [12]. In addition, infection with enteric pathogens can impair the tight junctions of IECs, resulting in the dysfunction of intestinal homeostasis [8]. It is reported that *S.* Typhimurium disrupted the tight junctions in IECs, contributing to its invasion and spread, and leading to an intense inflammatory response [13]. However, the mechanism of *S*. Typhimurium invasion remains to be further studied. This study established an IPEC-J2 cell model of *S.* Typhimurium infection to assess the inflammatory response and injury induced by *S.* Typhimurium. At the same time, we probed the positive effect of *L. johnsonii* L531 in anti-inflammation and protection of the intestinal barrier. We assumed that *L. johnsonii* L531 pre-incubation attenuated *S.* Typhimurium-induced inflammatory response by modulating TLR4/NF-κB/NLRP3 inflammasome signaling pathway.

## 2. Materials and Methods

### 2.1. Chemicals and Antibodies

The following chemicals in this study were purchased from Solarbio (Beijing, China): phenylmethanesulfonyl fluoride (PMSF, P0100), Radio Immunoprecipitation Assay (RIPA) buffer (R0010), Protease Inhibitor Cocktail (539133), SDS-PAGE loading buffer (P1040), phosphate buffered solution with Tween 20 (PBST, P1033), Albumin Bovine (BSA, A8020), 4′,6-diamidino-2-phenylindole (DAPI, C0065), 4% paraformaldehyde (PFA, P1110), Mounting Medium, antifading (S2100), and neutral balsam (G8590).

The relevant inhibitors in this study were purchased from MCE (Danvers, MA, USA): Resatorvid (HY-11109), pyrrolidinedithiocarbamate ammonium (APDC, HY-18738), and MCC950 (HY-12815A); while lipopolysaccharide (LPS, S1732) was purchased as an activator from Beyotime (Shanghai, China).

The following antibodies were used as part of this study: TLR4 (WL00196), MyD88 (WL02494), NLRP3 (WL02635), ASC (WL02462) were purchased from Wanleibio (Shenyang, China); p-NF-κB p65 (AP0123) were purchased from Abclonal (Wuhan, China); NF-κB p65 (#6956) was purchased from CST (Danvers, MA, USA); p-IκB-α (bs-18129R) and IκB-α (bs-1287R) were purchased from Bioss (Beijing, China); the following primary antibodies were purchased from proteintech (Rosemont, IL, USA): ZO-1 (21773-1-AP), Occludin (27260-1-AP), Claudin-1 (13050-1-AP), Caspase-1 (22915-1-AP); β-actin (66009-1-Ig), GAPDH (60004-1-Ig), Tubulin (66031-1-Ig), Histone-H3 (17168-1-AP). The secondary antibody HRP-labeled goat anti-rabbit IgG (H + L) (PR30009) was purchased from proteintech (Rosemont, IL, USA), and the HRP-labeled goat anti-mouse IgG (H + L) (HS201-01) was purchased from TransGen (Beijing, China). In addition, the Nuclear and Cytoplasmic Protein Extraction Kit (P0027) was obtained from Beyotime (Shanghai, China).

### 2.2. Bacterial Strains and Growth Conditions

The bacterial strains used in this study included clinical isolates *Salmonella* Typhimurium strain and *Lactobacillus johnsonii* L531. The clinical isolates *Salmonella* Typhimurium strain (denoted *S.* Typhimurium) is one of the *Salmonella* Typhimurium strains that were obtained from feces samples or small intestinal contents from swine with diarrhea in China from 2014–2016 [4]. *Lactobacillus johnsonii* L531 (denoted *L. johnsonii* L531) was isolated from the intestinal contents of healthy weaned piglets in our previous work [14]. In order to allow the co-localization of *S.* Typhimurium and cells, *S.* Typhimurium was transfected with a plasmid encoding a green fluorescent protein reporter. *S.* Typhimurium was grown overnight at 37 °C with shaking (200 rpm) in LB/Amp^+^ (Land Bridge, Beijing, China) broth. *L. johnsonii* L531 was grown on De Man, Rogosa, and Sharpe (MRS, Land Bridge, Beijing, China) agar in a bio-bag-contained anaerobic incubator (MGC, Tokyo, Japan) at 37 °C for 48 h, and then, single colonies were selected and inoculated into the tube which was filled with MRS broth in the anaerobic incubator at 37 °C for 12 h.

### 2.3. Cell Culture

IPEC-J2 cells were grown in DME/F-12 1:1 (Cytiva, Marlborough, MA, USA) supplemented with 10% heat-inactivated fetal bovine serum (ThermoFish Scientific, Waltham, MA, USA) and 1% penicillin/streptomycin (ThermoFish Scientific, Waltham, MA, USA) at 37 °C with 5% CO_2_ and humidified (95%) atmosphere, which was regulated by a CO_2_ incubator (SANYO, Osaka, Japan).

For bacterial infections, IPEC-J2 Cells were seeded in 6-well (3 × 10^5^ cells/well) culture plates (CORNING, NY, USA) under four groups/conditions: (i) medium alone (donated CN); (ii) *L. johnsonii* L531 pre-incubation (multiplicity of infection, MOI = 3) for 3 h prior to exposure to *S.* Typhimurium (MOI = 15) (donated LS); (iii) *S.* Typhimurium alone (MOI = 15) (donated ST), or (iv) incubation with *L. johnsonii* L531 (MOI = 3) (donated L531) [7].

After being challenged by *S.* Typhimurium for 30 min, the cells were washed three times with phosphate buffer saline (1×) (PBS, Cytiva, Marlborough, MA, USA) containing gentamycin (100 μg/mL, Sangon Biotech, Shanghai, China); cells were, then, treated with gentamicin (100 μg/mL) for 2 h to kill extracellular bacteria. Next, the infected cells were cultured in a medium with low concentration gentamicin (10 μg/mL) for the next 4 h. Finally, cells were harvested for our follow-up experiments [15].

In this study, we used some inhibitors and an activator. Before infections, Resatorvid (10 mM) and APDC (50 mM) were incubated with cells for 6 h, and MCC950 (100 mM) was incubated for 30 min, while LPS (2 mg/mL) was incubated for 12 h.

### 2.4. Western Blotting

IPEC-J2 cells were lysed on ice using RIPA buffer containing 1% Protease Inhibitors Cocktail and PMSF. The lysates were sonicated and, then, centrifuged at 13,000 rpm for 15 min at 4 °C. Only supernatants were collected as protein samples. Protein concentrations were detected by using a BCA kit (ThermoFish Scientific, Waltham, MA, USA). Dilute the protein samples, with RIPA and SDS-PAGE loading buffer, to the same concentration (25 μg/μL). Equal amounts of samples were loaded in each lane. Different molecular weight proteins were separated by SDS-PAGE and electrophoretically transferred onto PVDF membranes (Millipore, Burlington, MA, USA). PVDF membranes were blocked with 5% skimmed milk and incubated with primary antibodies overnight at 4 °C. After being washed in PBST, PVDF membranes were hybridized with secondary antibodies at room temperature for one hour, followed by washing with PBST. In the end, Western blotting images were visualized on s Tanon-5200 Chemiluminescent Imaging System (Tanon, Shanghai, China).

### 2.5. Immunofluorescence

IPEC-J2 cells seeded on glass slips (Solarbio, Beijing, China) in 24-well culture plates. The coverslips were exposed to *L. johnsonii* L531 when the number of cells had increased to 60–70%. After 3 h, the cells were infected with *S.* Typhimurium as described above. After being laved three times with cold PBS, the IPEC-J2 cells were fixed by 4% PFA for 10 min, rinsed with PBS to remove PFA, then incubated for 12 min in 1% Triton X-100 (Aladdin, Shanghai, China). After being washed, cells were incubated in 3% BSA for blocking. The permeabilized cells were incubated with primary antibody overnight at 4 °C. Then, after being served with PBS again, cells were incubated with goat anti-rabbit/mouse IgG (H + L), fluorescein Isothiocyanate conjugate (TransGen, Beijing, China) for 1 h at room temperature and DAPI for 7 min. Cover the cell slides on the micro slides with Mounting Medium, antifading, and around the cell slides with neutral balsam to prevent drying. Specimens were examined with a Leica SP8 Laser Scanning confocal microscope (Leica, Wetzlar, Germany).

### 2.6. Real-Time Quantitative PCR

Total RNA was extracted from IPEC-J2 cells by using RNAiso Plus (Takara, San Jose, CA, USA). The final extract was resuspended in RNase-free water, and the RNA concentration and purity were measured with Nanodrop (ThermoFish Scientific, Waltham, MA, USA). Then, cDNA was generated by PrimeScript^TM^ reagent Kit with gDNA Eraser (Takara, San Jose, CA, USA) following the manufacturer’s protocol. The qRT-PCR primer sequences designed and synthesized by Sangon Biotech are listed in Table 1. The GAPDH gene was used as a standardized internal control. The 2^−ΔΔCT^ method was used to express the relevant gene mRNA expression data as a fold change [7].

### 2.7. Statistical Analysis

The statistical significance of differences between groups was assessed using one-way analysis of variance (ANOVA) with Bonferroni correction for multiple comparisons. Statistical differences were evaluated within individual experiments based on mean values from 3 independent samples per experimental condition and across at least two independently performed experiments. *p* values greater than or equal to 0.05 were considered statistically significant. All analyses were performed using Prism v7.0 software (GraphPad, San Diego, CA, USA).

## 3. Results

### 3.1. L. johnsonii L531 Attenuates S. Typhimurium-Induced Inflammatory Response in IPEC-J2 Cells

**Figure 1:**
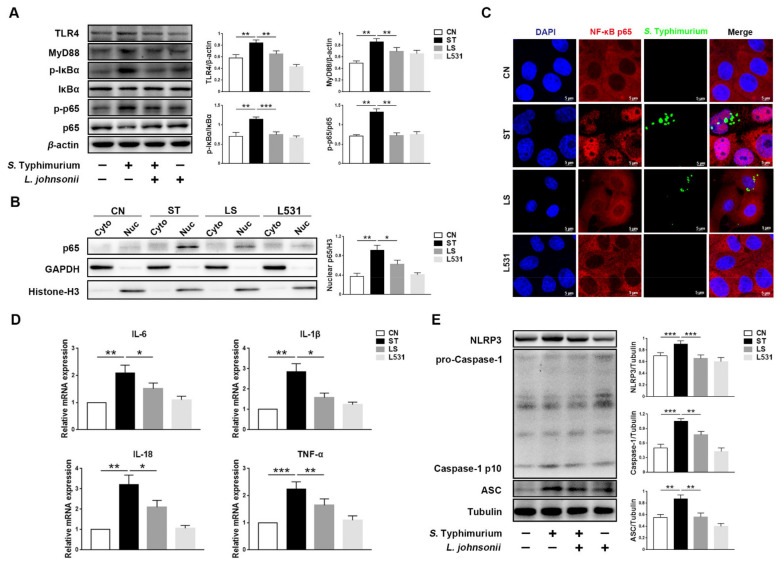
*L. johnsonii* L531 attenuates *S.* Typhimurium-induced inflammatory response in IPEC-J2 cells. (**A**) Western blotting of TLR4, MyD88, p-IκBα, IκBα, p-p65, and p65. (**B**) Western blotting of cytoplasmic and nuclear p65. (**C**) Representative immunofluorescence images of *S.* Typhimurium (green), NF-κB p65 (red), DAPI (blue) stains the nucleus. Scale bar, 5 µm. (**D**) Pro-inflammatory cytokines mRNA expression levels in IPEC-J2 cells were detected by qRT-PCR. (**E**) Western blotting of NLRP3, ASC, Caspase-1. CN (Control), ST (*S.* Typhimurium), LS (*L. johnsonii* L531 + *S.* Typhimurium), L531 (*L. johnsonii* L531), Cyto (Cytoplasm), Nuc (Nucleus). The panels show proteins or cytokines quantification using ImageJ software (version 1.50). The values are shown as the mean ± SEM; *n* = 3. * *p* < 0.05, ** *p* < 0.01, *** *p* < 0.001.

To explore the relationships among *L. johnsonii* L531, *S.* Typhimurium, and inflammation, we measured the expression levels of inflammatory-associated proteins. The Western blotting analysis demonstrated that compared with the CN group, and increased proteins expression of TLR4, MyD88, p-IκBα, and p-p65 were observed in the ST group, but the increase in those proteins was less in the LS group (Figure 1A). In addition, nuclear p65 as an index to reflect the intensity of activation of the NF-κB pathway was measured. In the ST group, the protein expression of nuclear p65 was the highest in all groups, but the protein level was lower in the LS group (Figure 1B). The results of immunofluorescence images were consistent with the results of Western blotting; compared with the LS group, we observed a stronger co-localization of DAPI and NF-κB p65 in the ST group, whereas most of the p65 were detected in cytoplasm in the CN and the L531 group (Figure 1C). Furthermore, *S.* Typhimurium up-regulated the expression levels of pro-inflammatory cytokines, such as IL-6, IL-1β, IL-18, and TNF-α, whereas *L. johnsonii* L531 pre-incubation can diminish the expression of these factors (Figure 1D). In addition, *S.* Typhimurium increased the protein levels of NLRP3 and ASC and promoted the cleavage of Caspase-1, while *L. johnsonii* L531 pre-incubation hindered the above process (Figure 1E).

### 3.2. L. johnsonii L531 Ameliorates S. Typhimurium-Induced Damage of Tight Junctions in IPEC-J2 Cells

**Figure 2:**
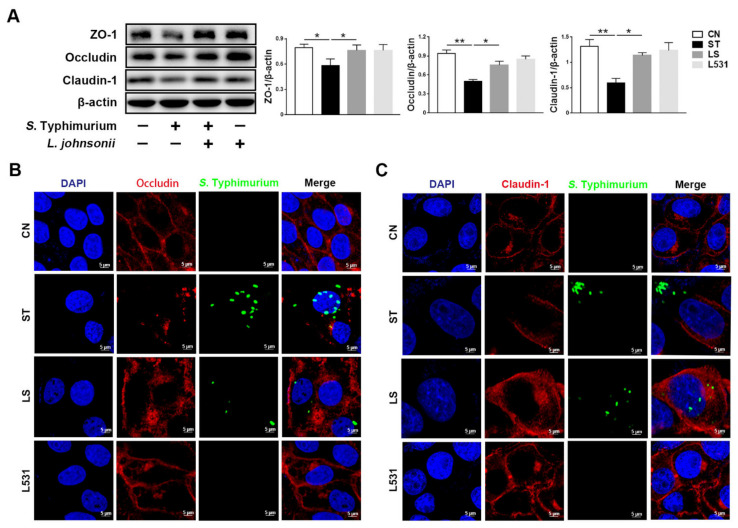
*L. johnsonii* L531 attenuates *S.* Typhimurium-induced damage of tight junctions in IPEC-J2 cells. (**A**) Western blotting of ZO-1, Occludin, Claudin-1. (**B**) Representative immunofluorescence images of *S.* Typhimurium (green), Occludin (red), DAPI (blue) stains the nucleus. Scale bar, 5 µm. (**C**) Representative immune-fluorescence images of *S.* Typhimurium (green), Claudin-1 (red), DAPI (blue) stains the nucleus. Scale bar, 5µm. CN (Control), ST (*S.* Typhimurium), LS (*L. johnsonii* L531 + *S.* Typhimurium), L531 (*L. johnsonii* L531). The panels show proteins quantification using ImageJ software (version 1.50). The values are shown as the mean ± SEM; *n* = 3. * *p* < 0.05, ** *p* < 0.01.

To probe the effects of *S.* Typhimurium and *L. johnsonii* L531 on intestinal tight junctions, we measured the expression of tight junction proteins ZO-1, Occludin, and Claudin-1. Western blotting analysis manifested that the expression levels of tight junction proteins ZO-1, Occludin, and Claudin-1 in the ST group were significantly lower than those in the CN and the L531 group. However, *L. johnsonii* L531 pre-incubation could observably alleviate *S.* Typhimurium-induced decrease in tight junction proteins (Figure 2A). Immunofluorescence images showed that *S.* Typhimurium destroyed the continuity of tight junctions, which is consistent with the results of Western blotting. In contrast, *L. johnsonii* L531 pre-incubation could memorably reduce the damage of *S.* Typhimurium to the integrity of tight junctions among IPEC-J2 cells and maintain the continuity of tight junctions (Figure 2B,C). In brief, *L. johnsonii* L531 can ameliorate *S.* Typhimurium-induced tight junctions damage.

### 3.3. L. johnsonii L531 Inhibits S. Typhimurium-Induced Assembly of the NLRP3 Inflammasome to Maintain Tight Junctions

**Figure 3:**
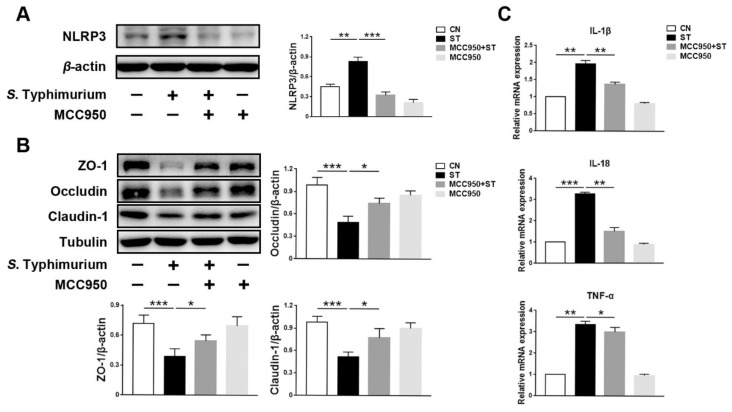
*L. johnsonii* L531 inhibits *S.* Typhimurium-induced assembly of the NLRP3 inflammasome to maintain tight junctions. (**A**) Western blotting of NLRP3. (**B**) Western blotting of ZO-1, Occludin, Claudin-1. (**C**) Pro-inflammatory cytokines mRNA expression levels in IPEC-J2 cells were detected by qRT-PCR. CN (Control), ST (*S.* Typhimurium). The panels show proteins or cytokines quantification using ImageJ software (version 1.50). The values are shown as the mean ± SEM; *n* = 3. * *p* < 0.05, ** *p* < 0.01, *** *p* < 0.001.

To evaluate the relationship between the NLRP3 inflammasome and tight junctions, we successfully suppressed the expression of NLRP3 (Figure 3A). Compared with the ST group, the expression levels of tight junction proteins ZO-1, Occludin, and Claudin-1 were higher in the MCC950 + ST and the MCC950 group (Figure 3B). Furthermore, decreased levels of pro-inflammatory cytokines IL-1β, IL-18, and TNF-α can be detected in the MCC950 + ST group (Figure 3C).

### 3.4. L. johnsonii L531 Inhibits S. Typhimurium-Induced Activation of NF-κB Pathway to Maintain Tight Junctions

**Figure 4:**
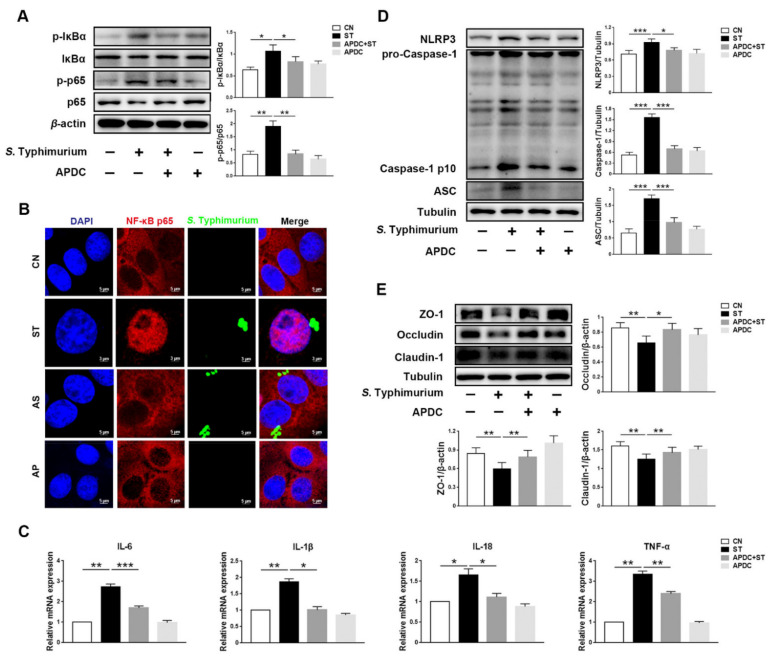
*L. johnsonii* L531 inhibits *S.* Typhimurium-induced activation of the NF-κB pathway to maintain tight junctions. (**A**) Western blotting of p-IκBα, IκBα, p-p65, and p65. (**B**) Representative immunofluorescence images of *S.* Typhimurium (green), NF-κB p65 (red), DAPI (blue) stains the nucleus. Scale bar, 5 µm. (**C**) Pro-inflammatory cytokines mRNA expression levels in IPEC-J2 cells were detected by qRT-PCR. CN control, ST *S.* Typhimurium. (**D**) Western blotting of NLRP3, ASC, Caspase-1. (**E**) Western blotting of ZO-1, Occludin, Claudin-1. The panels show proteins or cytokines quantification using ImageJ software (version 1.50). The values are shown as the mean ± SEM; *n* = 3. * *p* < 0.05, ** *p* < 0.01, *** *p* < 0.001.

The Western blotting analysis demonstrated that compared with the ST group, the APDC + ST group significantly reduced the phosphorylation of NF-κB pathway-related proteins p65 and IκBα (Figure 4A). Furthermore, the results of immunofluorescence images indicated no significant difference in the nuclear p65 between the CN and the APDC + ST group (Figure 4B), indicating that *S.* Typhimurium-induced activation of the NF-κB pathway was fully inhibited. In addition, the expression levels of inflammatory cytokines IL-6, IL-1β, IL-18, and TNF-α were reduced in the APDC + ST group (Figure 4C). Simultaneously, the results of Western blotting showed that compared with the ST group, the expression levels of NLRP3, ASC, and Caspase-1 p10 were lower, which indicated the activation of NLRP3 was limited in the APDC + ST group (Figure 4D). Of note, compared with the CN group, no significance was found in the expression levels of tight junction proteins ZO-1, Occludin, and Claudin-1 in the APDC + ST group (Figure 4E).

### 3.5. L. johnsonii L531 Inhibits S. Typhimurium-Induced Activation of TLR4 Pathway to Maintain Tight Junctions

**Figure 5:**
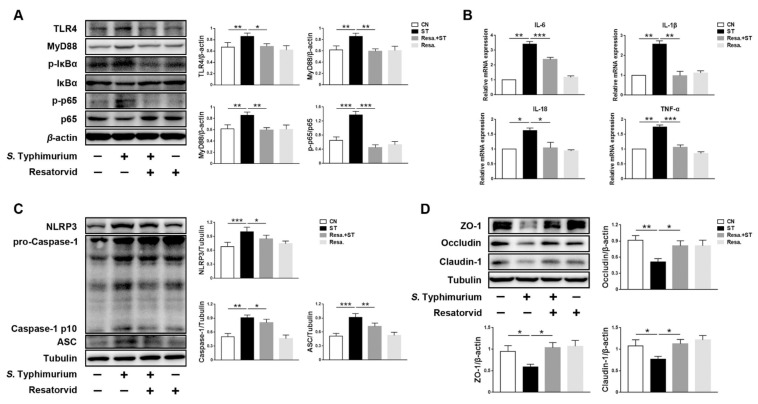
*L. johnsonii* L531 inhibits *S.* Typhimurium-induced activation of TLR4 pathway to maintain tight junctions. (**A**) Western blotting of TLR4, MyD88, p-IκBα, IκBα, p-p65, and p65. (**B**) Pro-inflammatory cytokines mRNA expression levels in IPEC-J2 cells were detected by qRT-PCR. (**C**) Western blotting of NLRP3, ASC, Caspase-1. (**D**) Western blotting of ZO-1, Occludin, Claudin-1. CN (Control), ST (*S.* Typhimurium), Resa. (Resatorvid). The panels show proteins or cytokines quantification using ImageJ software (version 1.50). The values are shown as the mean ± SEM; *n* = 3. * *p* < 0.05, ** *p* < 0.01, *** *p* < 0.001.

Western blotting analysis revealed that the expression levels of TLR4 and MyD88 in the Resa. + ST group was similar to those in the CN group, indicating that the TLR4/MyD88 pathway was inhibited. Furthermore, compared with the ST group, p-IκBα and p-p65, the NF-κB pathway associated proteins, were lower in the Resa. + ST group (Figure 5A). Meanwhile, the expression levels of pro-inflammatory cytokines IL-6, IL-1β, IL-18, and TNF-α were lower in the RS group (Figure 5B). In addition, the expression levels of NLRP3, ASC, and Caspase-1 p10 were reduced in the Resa. + ST group (Figure 5C). On the contrary, the protein levels of tight junction proteins ZO-1, Occludin, and Claudin-1 were higher in the Resa. + ST group (Figure 5D).

### 3.6. L. johnsonii L531 Alleviates LPS-Induced Inflammatory Response in IPEC-J2 Cells

**Figure 6:**
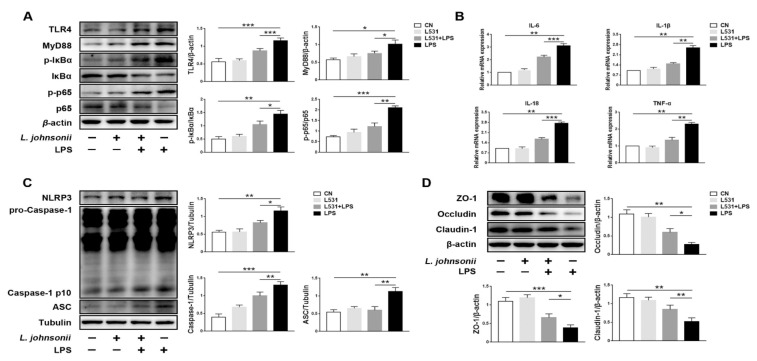
*L. johnsonii* L531 alleviates LPS-induced inflammatory response in IPEC-J2 cells. (**A**) Western blotting of TLR4, MyD88, p-IκBα, IκBα, p-p65, and p65. (**B**) Pro-inflammatory cytokines mRNA expression levels in IPEC-J2 cells were detected by qRT-PCR. (**C**) Western blotting of NLRP3, ASC, Caspase-1. (**D**) Western blotting of ZO-1, Occludin, Claudin-1. CN (Control), L531 (*L. johnsonii* L531), LPS (lipopolysaccharide). The panels show proteins or cytokines quantification using ImageJ software (version 1.50). The values are shown as the mean ± SEM; *n* = 3. * *p* < 0.05, ** *p* < 0.01, *** *p* < 0.001.

Compared with the LPS group, decreased proteins expression levels of TLR4 and MyD88 were observed in the L531 + LPS group. Meanwhile, the expression levels of p-IκBα and p-p65 were lower as well (Figure 6A). In addition, the expression levels of pro-inflammatory cytokines IL-6, IL-1β, IL-18, and TNF-α have experienced a significant decline in the L531 + LPS group (Figure 6B). It was worth noting that LPS up-regulated the protein levels of NLRP3, ASC, and pro-Caspase-1 (Figure 6C), and it also damaged the integrity of tight junctions (Figure 6D). Conversely, *L. johnsonii* L531 can remarkably retard this trend (Figure 6C,D).

## 4. Discussion

*S.* Typhimurium is an invasive enteric pathogen capable of stimulating intense intestinal inflammation [16]. Aberrant intestinal inflammation results in the availability of nutrients, allowing *S.* Typhimurium to gain a growth advantage in competition with intestinal flora [17]. Besides, *S.* Typhimurium modulates tight junction proteins to break through the intestinal epithelial barrier, thereby accelerating their translocation and transmission [18,19]. Therefore, we explored the relationship between tight junctions and *S.* Typhimurium-induced inflammatory response. In this study, *S.* Typhimurium elicited severe inflammatory response via three signaling pathways involving TLR4, NF-κB, and NLRP3 inflammasome. Meanwhile, a significant increase in the expression levels of IL-6, IL-1β, IL-18, and TNF-α could be detected. Furthermore, immunofluorescence images showed that the intact structure of tight junctions became discontinuous, which indicated *S.* Typhimurium induced extreme damage to tight junctions ZO-1, Occludin, and Claudin-1.

Multiple reports have demonstrated that proper probiotic supplementation can reverse intestinal damage and reduce inflammation [20,21]. In our study, *L. johnsonii* L531 pre-incubation inhibited the activation of TLR4/MyD88, NF-κB, and NLRP3 inflammasome signaling pathways, as well as suppressed the expression levels of pro-inflammatory cytokines, including IL-6, IL-1β, IL-18, and TNF-α. Intestinal commensal bacteria have a protective effect on the intestinal epithelial barrier by maintaining the physiological integrity of tight junctions that sustain their barrier function during enteric pathogens challenges [22]. Our data showed that *L. johnsonii* L531, as a pig-derived probiotic, successfully maintained the integrity of tight junctions in IPEC-J2 cells after the *S.* Typhimurium challenge. Notably, the magnitude of the inflammatory response was inversely correlated with the integrity of the tight junctions.

The NLRP3 inflammasome, an intracellular sensor, is vital for host immune defenses against pathogenic bacteria. However, over-expression of the NLRP3 leads to inflammatory diseases [12]. Studies demonstrated that inhibition of the NLRP3 inflammasome could alleviate endothelial gap junction dysfunction [23]. In this study, we confirmed that inhibiting the assembly of the NLRP3 inflammasome reduced the expression levels of the associated pro-inflammatory cytokines IL-1β/IL-18 and mitigated tight junction injury induced by *S.* Typhimurium. The above results indicated that tight junction proteins are closely associated with the NLRP3 inflammasome. The NLRP3 inflammasome pathway can be regulated by the upstream nuclear factor-kappa B (NF-κB) signaling pathway [11] and can also be directly activated by Caspases (Caspase-4 and Caspase-5 in humans, and Caspase-11 in mouse), which sense cytosolic LPS [24]. In this study, we found that the *S.* Typhimurium-induced NLRP3 inflammasome signaling pathway is strictly regulated by the NF-κB signaling pathway. Inhibiting the NF-κB signaling pathway by Resatorvid impeded the expression of NLRP3, the adaptor protein ASC, and pro-Caspase-1 suggested that *S.* Typhimurium activated the assembly of the NLRP3 inflammasome via the NF-κB-dependent pathway. We suspected that *S.* Typhimurium forms Salmonella-containing vacuole while invading IPEC-J2 cells, thereby blocking the sensing of *S.* Typhimurium LPS by intracellular receptors.

NF-κB signaling pathway is a crucial mediator of stimulating signals and cytokines and plays the most critical role in the inflammatory response [25]. It is reported that the NF-κB signaling pathway is significantly activated after the *S.* Typhimurium challenge [13]. As the data showed, *S.* Typhimurium invasion resulted in IκBα and p65 phosphorylation. It has been reported that NF-κB dimers bind to IκB proteins and remain in the cytoplasm at its inactive stage. However, rapid degradation of IκBα during activation of the NF-κB signaling pathway leads to exposure of the nuclear localization sequence of p65 and enhances the nuclear translocation of the NF-κB dimers (p65/p50) [26]. In this study, we used two methods to detect the nuclear p65. The results of Western blotting showed a large number of nuclear p65 in the ST group. Meanwhile, immunofluorescence images manifested a stronger co-localization of DAPI and p65 after the *S.* Typhimurium challenge. The above results indicated that *S.* Typhimurium strongly activated the NF-κB pathway. Studies showed that reducing p65 nuclear translocation effectively inhibits the transcription and expression levels of inflammatory cytokine genes involved in the NF-κB pathway [27]. In this study, *L. johnsonii* L531 pre-incubation effectively slowed down the tendency of nuclear translocation of p65, which in turn suppressed the activation of NF-κB inflammatory pathways and inhibited the expression of associated factors. Compared with APDC (NF-κB inhibitor), regulation of NF-κB by *L. johnsonii* L531 was more modest or limited, but this was sufficient to mitigate tight junctions damage by *S.* Typhimurium.

Recently, studies showed that *S.* Typhimurium is able to bypass the innate immune receptors, directly activate the NF-κB pathway by effector proteins of its type III secretion systems, and then, induce the expression of the related genes [19]. However, we observed a significant increase in the expression of TLR4/MyD88, implying TLR4 could still recognize the invasion of *S.* Typhimurium. We, then, explored the role of TLR4 after the *S.* Typhimurium challenge. TLR4, as a transmembrane protein located on the cell surface, is the first to sense extracellular stimuli and quickly transmit signals to the cell [18], which makes it become an ideal signal transfer hub for *L. johnsonii* L531 to regulate the downstream pathways. After all, *L. johnsonii* L531, as an extracellular bacterium, apparently does not directly regulate the NF-κB pathway. In this study, *L. johnsonii* L531 down-regulated the expression of TLR4/MyD88 that inhibited the activation of TLR4/MyD88 pathway. To interrogate the regulation of TLR4 by *L. johnsonii* L531, we selected lipopolysaccharide (LPS) as the stimulus. Our data showed that *L. johnsonii* L531 alleviated the LPS-induced activation of TLR4, resulting in suppression of NF-κB and NLRP3 signaling pathways. Many studies have shown that LPS decreased the expression levels of tight junction proteins, such as ZO-1, Occludin, and Claudin-1 [28,29]. In our study, *L. johnsonii* L531 ameliorated LPS-induced tight junction injury demonstrated by steady expression levels of ZO-1, Occludin, and Claudin-1. To sum up, *L. johnsonii* L531 exploited TLR4/MyD88-dependent innate immune responses to alleviate *S.* Typhimurium-induced activation of the inflammatory signaling pathway.

## 5. Conclusions

The results of the study indicated that *S.* Typhimurium enhances inflammatory response by activating the TLR4/NF-κB/NLRP3 inflammasome signaling pathway in IPEC-J2 cells, thereby reducing the expression levels of tight junction proteins ZO-1, Occludin, and Claudin-1. On the contrary, *L. johnsonii* L531 pre-incubation successfully inhibited the excessive activation of the inflammatory signaling pathway described above and reduced the overexpression of inflammatory cytokines, thus contributing to maintaining the integrity of tight junctions. Our study proved the anti-inflammatory effects of *L. johnsonii* L531 and provided the theoretical support for *L. johnsonii* L531 as a probiotic to resist the damage of *S.* Typhimurium and showed a promising application prospect in the swine industry.

## Figures and Tables

**Table 1:** Information of oligonucleotide primers used for real-time qPCR.

Gene Product	Primer		GenBank Accession
	Direction	Sequences (5′ to 3′)	
IL-6	F	GGGAAATGTGGAGGCTGTG	NM_214399
	R	AGGGGTGGAGGCTTTGTCT	
IL-1β	F	GGCCGCCAAGGTATAATGA	NM_214055
	R	GGACCTCTGGGCATGGCTTTC	
IL-18	F	GCTGCTGAACGGGAAGACAA	NM_213997.1
	R	AAACACGGCTAGATGTCCCT	
TNF-α	F	GCCCACGTTGTGGCCAATGTCAAA	NM_214002
	R	GTTGTCTTTCAGGTTCACGCCGTT	
GAPDH	F	GCTGCTGAACGGGAAGACAA	NM_001206359
	R	AAACACGGCTAGATGTCCCT	

IL = interleukin; TNF-α = tumor necrosis factor-α; GAPDH = glyceraldehyde-3-phosphate dehydrogenase; F = forward; R = reverse.

## Data Availability

Data are available upon reasonable request from the corresponding author.
